# Receptor-Tyrosine Kinase Inhibitor Ponatinib Inhibits Meningioma Growth In Vitro and In Vivo

**DOI:** 10.3390/cancers13235898

**Published:** 2021-11-24

**Authors:** Tao Yu, Junguo Cao, Montadar Alaa Eddine, Mahmoud Moustafa, Andreas Mock, Cihan Erkut, Amir Abdollahi, Rolf Warta, Andreas Unterberg, Christel Herold-Mende, Gerhard Jungwirth

**Affiliations:** 1 Department of Neurosurgery, Division of Experimental Neurosurgery, University of Heidelberg, Im Neuenheimer Feld 400, 69120 Heidelberg, Germany; tao.yu@stud.uni-heidelberg.de (T.Y.); junguo.cao@med.uni-heidelberg.de (J.C.); mohammedmontadar.alaaeddine@med.uni-heidelberg.de (M.A.E.); andreas.mock@med.uni-heidelberg.de (A.M.); rolf.warta@med.uni-heidelberg.de (R.W.); andreas.unterberg@med.uni-heidelberg.de (A.U.); christel.herold-mende@med.uni-heidelberg.de (C.H.-M.); 2 Department of Radiation Oncology, University of Heidelberg, Im Neuenheimer Feld 400, 69120 Heidelberg, Germany; mahmoud.moustafa@med.uni-heidelberg.de (M.M.); a.amir@dkfz-heidelberg.de (A.A.); 3 Department of Clinical Pathology, Suez Canal University, 4.5 Km the Ring Road, Ismailia 41522, Egypt; 4 National Center for Tumor Diseases (NCT) Heidelberg, Department of Medical Oncology, Heidelberg University Hospital, Im Neuenheimer Feld 400, 69120 Heidelberg, Germany; 5 National Center for Tumor Diseases (NCT) Heidelberg, Department of Translational Medical Oncology, German Cancer Research Center (DKFZ), Im Neuenheimer Feld 581, 69120 Heidelberg, Germany; 6 Division of Applied Functional Genomics, German Cancer Research Center (DKFZ), Im Neuenheimer Feld 581, 69120 Heidelberg, Germany; cihan.erkut@nct-heidelberg.de

**Keywords:** meningioma, ponatinib, RTKi, NCH93, PDGFRA, PDGFRB, mitochondrial dysfunction

## Abstract

**Simple Summary:**

The clinical management for aggressive meningiomas remains challenging due to the lack of systemic treatment options. Receptor tyrosine kinases (RTKs) are frequently overexpressed in meningiomas and are associated with poor patient survival. In this study, we evaluated the clinically approved pan-RTK inhibitor ponatinib as a novel candidate for the treatment of aggressive meningiomas. Ponatinib decreased cell viability and proliferation of meningioma cells and subsequently induced programmed cell death. Furthermore, the drug demonstrated a considerable tumor growth inhibition without causing any adverse effects in mice. Mechanistically, this was presumably caused by blocking the PDGFR signaling pathway accompanied by induction of mitochondrial dysfunction. Altogether, the multi-RTKi ponatinib may serve as a promising candidate for targeted therapy for aggressive meningiomas.

**Abstract:**

To date, there is no standard-of-care systemic therapy for the treatment of aggressive meningiomas. Receptor tyrosine kinases (RTK) are frequently expressed in aggressive meningiomas and are associated with poor survival. Ponatinib is a FDA- and EMA-approved RTK inhibitor and its efficacy in meningioma has not been studied so far. Therefore, we investigated ponatinib as a potential drug candidate against meningioma. Cell viability and cell proliferation of ponatinib-treated meningioma cells were assessed using crystal violet assay, manual counting and BrdU assay. Treated meningioma cell lines were subjected to flow cytometry to evaluate the effects on cell cycle and apoptosis. Meningioma-bearing mice were treated with ponatinib to examine antitumor effects in vivo. qPCR was performed to assess the mRNA levels of tyrosine kinase receptors after ponatinib treatment. Full-length cDNA sequencing was carried out to assess differential gene expression. IC50 values of ponatinib were between 171.2 and 341.9 nM in three meningioma cell lines. Ponatinib induced G0/G1 cell cycle arrest and subsequently led to an accumulation of cells in the subG1-phase. A significant induction of apoptosis was observed in vitro. In vivo, ponatinib inhibited meningioma growth by 72.6%. Mechanistically, this was associated with downregulation of PDGFRA/B and FLT3 mRNA levels, and mitochondrial dysfunction. Taken together, ponatinib is a promising candidate for targeted therapy in the treatment of aggressive meningioma.

## 1. Introduction

Meningioma (MGM) is the most frequent primary brain tumor, accounting for 38.3% of primary brain tumors overall reported in the United States between 2013 and 2017 [1]. Approximately 80% of MGMs are classified into World Health Organization (WHO) grade I (benign), 15–20% in WHO grade II (atypical), and 1–4% in grade III (malignant) meningiomas [1]. The majority of MGMs display an indolent clinical course, with a 5–10% recurrence rate at 5 years [1]. However, high-grade MGMs are associated with a higher recurrence rate and poor prognosis [1]. In recent studies, median survival for malignant MGM was 53 months while 10-year survival was only 23% to 59.6% [1,2]. The mainstay of therapy consists of surgical removal of the tumor and/or radiotherapy. However, there is currently no effective systemic treatment available for the treatment of aggressive meningiomas [3,4].

Establishing novel drug-based therapies for meningiomas is essentially based on the understanding of key molecular drivers for meningioma growth and tumor invasion. In the recent decade, several frequent genetic alterations were discovered, which led to the subsequent initiation of clinical studies (NCT02523014; NCT03071874) [5,6,7,8,9]. However, mutated targets are mainly found in non-NF2 benign meningiomas [5,7]. Several studies have demonstrated that key signaling pathways such as phosphoinositide 3-kinase (PI3K) and mitogen-activated protein kinase (MAPK) are activated in both benign and higher grade meningiomas [10,11,12,13]. These signaling pathways are activated by the receptor tyrosine kinase family (RTKs), such as epidermal growth factor receptor (EGFR), fibroblast growth factor receptor (FGFR), platelet-derived growth factor receptor (PDGFR), vascular endothelial growth factor receptor (VEGFR), or insulin-like growth factor receptor (IGFR) [13]. Moreover, expression of VEGFR, PDGFR, and EGFR is associated with high-grade MGMs and shorter progression-free survival (PFS) [14,15,16]. Therefore, targeting RTKs using tyrosine kinase inhibitors (TKis) emerged as a promising approach for the treatment of MGMs [17]. Previously, several studies investigated anti-meningioma effects of TKis [18,19,20]. Despite a previous preclinical analysis of regorafenib and sorafenib showed antitumor activity in vitro [19], results from clinical trials of TKi-treated meningioma showed no to only a moderate response. For example, a phase II trial of another TKi, imatinib, showed 0% of 6-month progression-free survival (6M PFS) for atypical and malignant MGMs [18]. In addition, EGFRi erlotinib and gefitinib resulted in 29% of 6M PFS without any objective imaging responses for the treatment of atypical and malignant MGMs in a recent phase II trial [20]. One possible explanation of the rather modest clinical activity of TKis might be drug resistance via bypass activation of other RTKs ensuring constant activation of the downstream signaling [21]. This mechanism is currently best understood in lung cancer [21]. Therefore, TKis with high affinity to multiple RTKs may be a promising approach for targeted therapy of high-grade MGMs. Sunitinib inhibiting PDGFR, VEGFR, and KIT is such an example. The drug has been evaluated in a phase II clinical trial with a 6M PFS of 42% in recurrent and progressive atypical and anaplastic MGMs [22,23]. Although results were promising, the small sample size and a considerable toxicity to sunitinib were major limitations of this study [23].

Mitochondria are intracellular double-layer organelles generating more than 95% of energy in both normal and cancer cells [24]. Therefore, dysfunction of mitochondria by any cause may induce apoptosis and/or necrosis [25]. Interestingly, several studies reported that TKis may disrupt the normal function of mitochondria in cancer by promoting reactive oxygen species (ROS) generation, and inducing mitochondrial fragmentation, mitophagy, and release of cytochrome c [25,26]. Furthermore, TKi-induced mitochondrial dysfunction is also associated with several adverse effects including diarrhea, fatigue, and hypertension [27]. Ponatinib is a FDA- and EMA-approved TKi for patients suffering from imatinib-resistant chronic myeloid leukemia (CML) and Philadelphia chromosome-positive (Ph+) acute lymphoblastic leukemia [28]. Beside its potent inhibition of fusion gene Bcr-Abl, ponatinib also inhibits multiple other RTKs, including PDGFR, VEGFR1 and 2, FGFR1–4, RET, c-KIT, and FMS-like tyrosine kinase 3 (FLT3) [29,30,31,32]. Moreover, ponatinib demonstrated potent antitumor activity in preclinical models of a variety of human cancers, including rhabdomyosarcoma, thyroid cancer, lung cancer, gastrointestinal stromal tumors, glioblastoma, and endometrial cancer [29,33,34,35,36,37]. In this study, we evaluated the efficacy of the ponatinib in MGM cells and found that ponatinib demonstrated potent anti-meningioma effects in vitro and in vivo presumably through PDGFRA/B inhibition and mitochondrial dysfunction.

## 2. Materials and Methods

### 2.1. Cell Culture

The benign cell line Ben-Men-1 (Leibniz Institute DSMZ, Braunschweig, Germany), the anaplastic meningioma cell line NCH93 [10] and IOMM-Lee were cultured in Dulbecco’s Modified Eagle Medium (DMEM, Life Technologies Limited, Paisley, UK) supplemented with 10% fetal bovine serum superior (Sigma-Aldrich, St. Louis, MO, USA), 2% GlutaMAX^TM^-I (100X, Life Technologies Corporation, Grand Island, NY, USA), and 1% Penicillin/Streptomycin (Life Technologies Corporation, Grand Island, NY, USA) at 37 °C in a humidified environment with 5% CO_2_ atmosphere. Mycoplasma contamination was excluded by 4′,6-diamidino-2-phenylindole staining (DAPI, Life Technologies Corporation, Eugene, OR, USA). NCH93 cell line was authenticated by STR DNA profiling analysis (Leibniz Institute DSMZ, Braunschweig, Germany).

### 2.2. Crystal Violet Assay

Cells were seeded at a density of 5000 cells/well in a 96-well plate with 200 µL medium. On the next day, cells were treated with ponatinib (MedChemExpress, Monmouth Junction, NJ, USA) in 9 concentrations ranging from 1 to 5000 nM and incubated for 48 h. Then, medium was removed, and cells were washed with 100 μL Dulbecco’s Phosphate Buffered Saline (DPBS, Life Technologies Limited, Paisley, UK) once. Subsequently, 50 μL of 0.5% crystal violet (Sigma-Aldrich, St. Louis, MO, USA) was added into wells and cells were incubated for 15 min at room temperature (RT) on a shaker. Next, crystal violet was removed and cells were washed with Aqua (B.Braun, Melsungen, Germany). Plates were left to dry overnight in a fume hood. On the following day, 200 μL of methanol (Carl Roth GmbH, Karlsruhe, Germany) was added to dissolve crystal violet. The optical density was recorded at 555 nm using a microplate reader (Infinite F200 pro, Tecan GmbH, Grödig, Austria). IC50 values were calculated by nonlinear regression in GraphPad Prism (version 9.0.0, GraphPad, San Diego, CA, USA).

### 2.3. Growth Curves

A total of 1 × 10^5^ cells of Ben-Men-1 and IOMM-Lee, and 5 × 10^5^ cells of NCH93 were seeded in 6-well plates. The next day, cells were treated with DMSO control or ponatinib at IC50 and 10 × IC50. Single cell suspensions were harvested at 0, 24, and 48 h after treatment, and manually counted using a Neubauer chamber (Reichert, Buffalo, NY, USA). Data were normalized to the DMSO control group.

### 2.4. BrdU Assay

Cell proliferation was measured using the Cell Proliferation ELISA, BrdU Kit (Roche Diagnostics GmbH, Mannheim, Germany). NCH93, Ben-Men-1, and IOMM-Lee were seeded in 96-well plates at a density of 4000 cells/well. Cells were treated with ponatinib at 5 × IC50. On days 1, 2, and 3, 10 µL BrdU labeling solution was added to the cells at a final concentration of 10 µM and incubated for 2 h. Next, the medium was removed from the cells by tapping off. Cells were fixed with 200 µL per well FixDenat and incubated for an additional 30 min at RT. FixDenat was removed and 100 µL anti-BrdU-POD working solution per well was added and incubated for 90 min at RT. The antibody conjugate was removed, and wells were rinsed 3 times with 200 µL PBS. Thereafter, 100 µL substrate solution was added and incubated for an additional 10 min at RT. Then, absorbance was measured at 450 nm and reference wavelength 690 nm by a microplate reader (Infinite F200 pro, Tecan GmbH, Grödig, Austria).

### 2.5. Wound Healing Assay

A total of 8 × 10^5^ cells from each cell line were seeded in 6-well plates. After reaching 100% confluency, a straight gap was generated on the cell monolayer using a 100 μL pipette tip. Then, the cell monolayer was washed carefully with 1 mL DPBS to remove cell debris and thereafter 2 mL fresh medium was added. Ponatinib was added at the concentration of IC50. DMSO was used as control (Sigma-Aldrich, St. Louis, MO, USA). Images of the gap were taken at 0 and 12 h. Migrated areas of cells were calculated by ImageJ (version1.8.0, National Institutes of Health, Bethesda, MD, USA). The areas covered by migrating ponatinib-treated cells were normalized to the mean of the DMSO-treated group.

### 2.6. Cell Cycle Analysis

A total of 3 × 10^5^ cells were seeded in 6-well plates. On the next day, cells were treated with ponatinib at 10 × IC50 or DMSO control (Sigma-Aldrich, St. Louis, MO, USA) for 24, 48, and 72 h. At each time point, cells were harvested and fixed with 85% ice-cold ethanol (Carl Roth GmbH, Karlsruhe, Germany). After incubation for 30 min at 4 °C, cells were centrifuged at 300× *g* for 5 min at 4 °C and ethanol was carefully removed. Cells were then washed with 1 mL ice-cold DPBS twice. Thereafter, cells were resuspended in 500 μL DPBS and 5 μL RNAse A (Lucigen, Middleton, WI, USA) was added to the cells and incubated for 30 min at 4° C. Prior to measurement, 1 µL propidium iodide (PI) (Sigma-Aldrich, St. Louis, MO, USA) was added at a final concentration of 50 µg/mL. Cells were analyzed by LSR II flow cytometer (BD Biosciences, San Jose, CA, USA) and cell cycle distribution was analyzed using the software FlowJo (version 10.6.1, FlowJo LLC, Ashland, OR, USA).

### 2.7. Apoptosis Assay

A total of 3 × 10^5^ cells were seeded in 6-well plates. On the following day, cells were treated with ponatinib at 10 × IC50 or DMSO control (Sigma-Aldrich, St. Louis, MO, USA) for 24, 48, and 72 h, respectively. At each time point, cells were collected from each well and transferred into a 15 mL Falcon tube (Corning Science Mexico SA de CV, Tamaulipas, Mexico). Subsequently, cells were centrifuged at 300× *g* for 5 min at 4 °C. Then, the supernatant was carefully discarded and the cell pellets were resuspended and washed twice with 1 mL ice-cold DPBS. Thereafter, cells were washed with 1 mL 1 × annexin V binding buffer. After centrifugation, the supernatant was removed and the cell pellet was resuspended with 100 µL 1 × annexin V binding buffer. Finally, cells were stained with 2 µL annexin V FITC antibody (Biolegend, San Diego, CA, USA) and 50 µg/mL PI and incubated for 15 min in the dark at 4 °C. Before detection, 1 × annexin V binding buffer was added to the cells to a total volume of 500 μL. Cells were then measured by flow cytometry within 1 h and analyzed using the software FlowJo (version 10.6.1, FlowJo LLC, Ashland, OR, USA).

### 2.8. Meningioma Xenograft Experiments

All experiments were done in accordance with the regulations of animal protection and approved by the Regierungspraesidium Karlsruhe, Germany. Five to six-week-old female NMRI/nu mice (Janvier Laboratory, Le Genest-Saint-Isle, France) received subcutaneous injections into the right flank with 4 × 10^6^ NCH93 cells in 100 µL medium and 100 µL Matrigel (Corning, San Diego, CA, USA). Tumor volume was measured daily by using a digital caliper and it was calculated using the following equation: Volume = (Length × Width^2^)/2 [38], where length represents the longest tumor diameter and width represents the perpendicular tumor diameter. When the tumor volume reached 200 mm^3^, mice were randomly divided into DMSO and ponatinib treatment groups. The dosage of 10 mg per kg bodyweight of ponatinib was administered intraperitoneally daily. The weight of the mice was measured on a daily basis. On day 21, mice were sacrificed, and blood was drawn and immediately sent to the central laboratory of University Hospital Heidelberg for further analysis. Excised tumors were weighed and photographed. Tumor tissues were snap-frozen and stored at −80 °C until further processing.

### 2.9. Immunohistochemical Staining

Acetone-fixed cryosection slides were prepared. Slides were incubated with rabbit polyclonal anti-Ki-67 (1:50 dilution, ab15580, Abcam, Cambridge, UK), cleaved caspase-3 (1:400 dilution, 9661, Cell Signaling, Danvers, MA, USA) antibodies diluted with DAKO diluent (Agilent Technologies, Santa Clara, CA, USA) for 60 min at RT, and washed three times with PBS-Tween-20 (0.05%) (Sigma-Aldrich, St. Louis, MO, USA). Next, secondary antibody (anti-rabbit, Vector, Burlingame, CA, USA) diluted in goat serum (Vector, Burlingame, CA, USA) and DPBS was applied to the slides and incubated for 30 min. After washing steps as described above, an avidin-biotin-complex (Vector, Burlingame, CA, USA) was added to the slides for 30 min incubation. Then, the AEC substrate (Vector, Burlingame, CA, USA) was applied to the slides and incubated for 3 min (anti-Ki-67) or 8 min (cleaved caspase-3). Finally, slides were counterstained with hematoxylin (Carl Roth GmbH, Karlsruhe, Germany) for 7 min. Ki-67 and cleaved caspase-3 positive cells were counted in ten high-power fields per slide. Rabbit IgGs (ab37415, Abcam, Cambridge, UK) served as a negative control.

### 2.10. Quantitative Real-Time PCR

Total RNA was extracted from eight xenograft tumor samples (four from the ponatinib-treated group and four from the DMSO-treated group) using the RNeasy Mini Kit (Qiagen, Valencia, CA, USA) according to the manufacturer’s instructions. RNA was quantified by NanoDrop ND-1000 spectrophotometer (Thermo-Scientific, Waltham, MA, USA). Equal amounts of total RNA (1 μg) were reverse-transcribed using the Transcriptor First Strand cDNA Synthesis Kit (Roche, Basel, Switzerland) with random hexamer primers for 1 h at 50 °C. qPCR was performed in quadruplicates on a LightCycler 480 (Roche, Basel, Switzerland) using the LightCycler 480 Probes Master and probes from the Universal Probe Library (Roche, Basel, Switzerland) as described (www.roche-applied-science.com). (last accessed on 5 February 2021). Relative fold changes between the expression of target genes were calculated by using the 2^−ΔΔCq^ method. *GAPDH*, *ACTB*, and *HPRT1* were used as reference genes. Relative expression mRNA levels of RTKs: *FLT3*, *FGFR1*, *FGFR2*, *FGFR4*, *PDGFRA*, *PDGFRB*, *VEGFR1*, and *VEGFR2* were normalized to the mean of the DMSO tumor samples. The primers used are listed in Table S1.

### 2.11. Full-Length cDNA Nanopore Sequencing

Fifty ng of total RNA from two ponatinib-treated and two control tumors were reverse transcribed and barcoded using the SQK-PCB109 kit (Oxford Nanopore Technology, Oxford, UK). Samples were run on a MinION R9.4.1 flowcell (Oxford Nanopore Technology, Oxford, UK). Base calling and demultiplexing were performed using Guppy 4.5.4. Filtered reads (minimum average Phred score 7) were aligned to a combined human and mouse genome (GRCh38.p13 and GRCm39) using minimap2 2.18, whereof only human gene (GENCODE.v37) alignments with a quality score more than 20 were counted using featureCounts 2.0.0. Differential gene expression analysis was performed using the package DESeq2 in the software R (v.4.1.0). Differentially expressed genes were determined based on a false discovery rate (FDR) cutoff of *p* < 0.05 and log2 fold change ≥1 or ≤−1.

### 2.12. Analysis of Mitochondrial Dysfunction

In order to explore functional differences between xenograft tumors, gene set enrichment analysis (GSEA) was performed using the packages clusterProfiler and enrichplot in the software R (v.4.1.0). For the identification of genes associated with mitochondrial dysfunction, differentially expressed genes were analyzed by GSEA using Gene Ontology (GO) gene sets accessed from MSigDB [39].

### 2.13. Statistical Analysis

All in vitro experiments were performed at least in triplicates and were independently repeated three times, and results were expressed as mean ± SEM. *p*-values were calculated using a two-tailed Student’s t-test in GraphPad (version 9.0.0, GraphPad, San Diego, CA, USA). *p*-values < 0.05 were considered significant (* *p* < 0.05; ** *p* < 0.01; *** *p* < 0.001).

## 3. Results

### 3.1. Ponatinib Decreased Viability and Proliferation of Meningioma Cells

**Figure 1:**
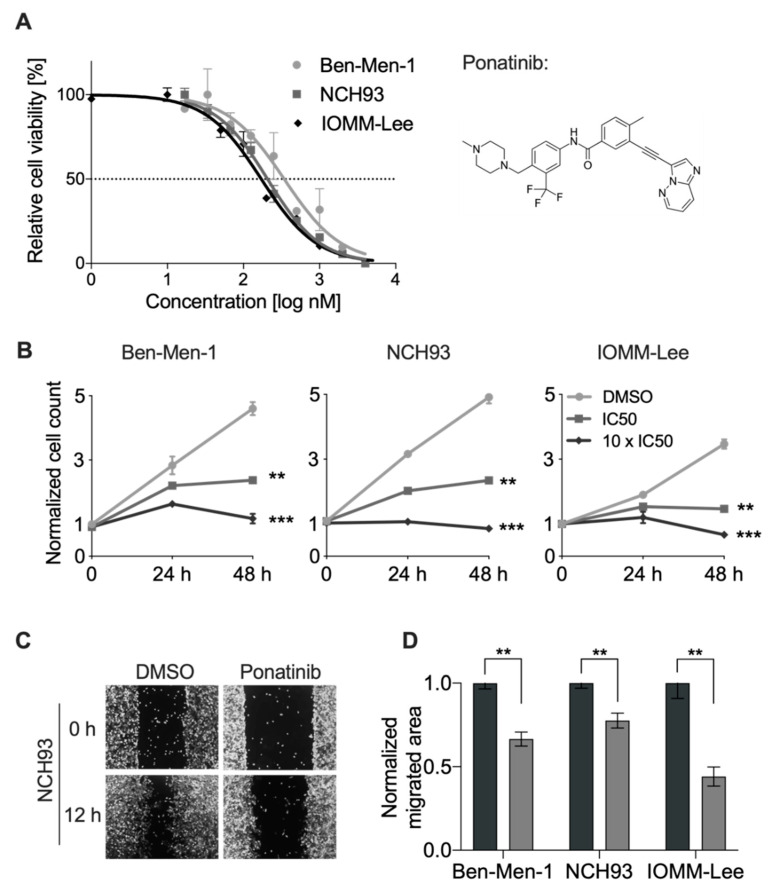
Ponatinib reduced meningioma cell proliferation and migration: (**A**) Illustrated are dose-curves of ponatinib in the benign meningioma cell line Ben-Men-1, and the malignant cell lines NCH93 and IOMM-Lee (left). The molecular structure of ponatinib is depicted (right). (**B**) Cells were treated with increasing concentrations (IC50 and 10 × IC50) of ponatinib or DMSO followed by a manual counting at 0 h, 24 h, and 48 h. (**C**) Wound healing assay using NCH93 cells showed delayed closure of the wound gap upon ponatinib treatment. (**D**) Quantification of the extent of the wound closure after 12 h. Each experiment was performed in triplicate and repeated three times. Results are expressed as mean ± SEM. ** *p* < 0.01; *** *p* < 0.001.

To investigate the efficacy of ponatinib for the treatment of meningioma, we assessed the half-maximal inhibitory concentration (IC50) of ponatinib using crystal violet assay in the anaplastic MGM cell lines IOMM-Lee, NCH93, and the benign MGM cell line Ben-Men-1 (Figure 1A). IC50 values of ponatinib ranged from 171.2 to 341.9 nM in all MGM cell lines, whereas IOMM-Lee presented with the lowest IC50 value of 171.2 nM (95% confidence interval: 147.2–199.0). Furthermore, the IC50 values of ponatinib in NCH93 and Ben-Men-1 were 206.7 nM (185.1–230.8) and 341.9 nM (250.1–467.5), respectively. To assess cell growth over time, cells were treated with ponatinib at IC50 and 10 × IC50 and the number of cells were counted manually. After 48 h treatment, the number of cells significantly decreased to 42.3%, 47.7%, and 51.6% compared to control in IOMM-Lee, NCH93, and Ben-Men-1, respectively (Figure 1B, *p* < 0.01). As expected, an even stronger inhibitory effect was observed when cells were treated with 10 × IC50 of ponatinib. The number of cells declined to 25.5%, 17.4%, and 19.2% compared to DMSO treatment, respectively (*p* < 0.001). To validate the effect of ponatinib on cell proliferation, we performed BrdU assay and treated cells with 5 × IC50 of ponatinib. Strikingly, incorporation of BrdU in ponatinib-treated cells was less than 0.4% compared to the control group in all cell lines after 24 h and continued until 72 h treatment (*p* < 0.001, Figure S1A). Collectively, ponatinib exhibited a strong antiproliferative effect on meningioma cell lines at the nanomolar level.

### 3.2. Ponatinib Reduced Migration of Meningioma Cells

To investigate the anti-migratory effect of ponatinib in meningioma cells, a wound healing assay was performed (Figure 1C). Twelve hours after treatment, the area covered by migrating cells was reduced by 55.8% in IOMM-Lee cells (*p* = 0.007, Figure 1D). To a lesser degree, treated NCH93 and Ben-Men-1 cells demonstrated a reduction of the area covered by migrating cells by 22.4% and 33.5%, respectively (*p* = 0.014 and *p* = 0.003, Figure 1D). These findings suggested that ponatinib is able to affect cell migration of benign and malignant meningioma cells.

### 3.3. Accumulation of Cells in G0/G1 and subG1-Phase upon Treatment

**Figure 2:**
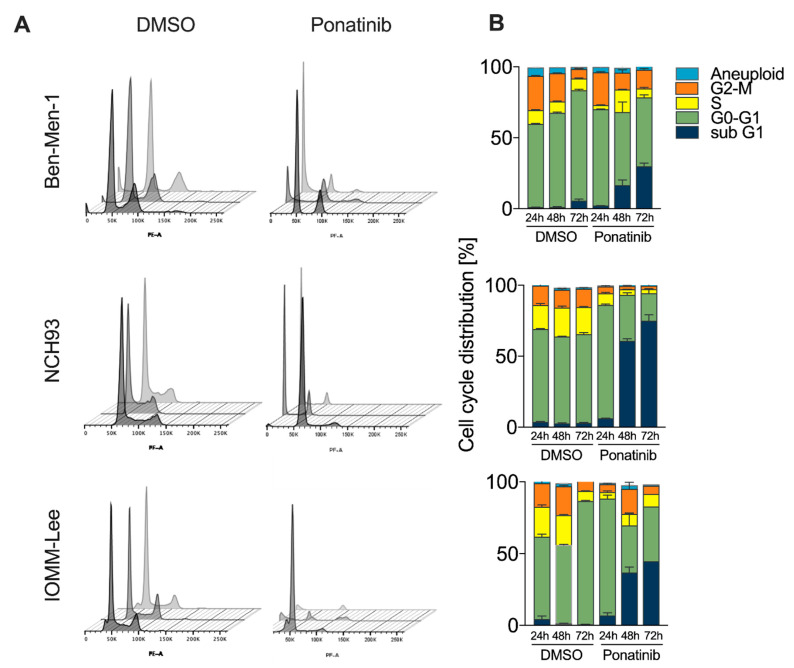
Ponatinib disrupts the meningioma cell cycle by induction of G0/1-arrest and subsequent accumulation of cells in subG1-phase: (**A**) Cell cycle distribution of meningioma cells treated with 10 × IC50 of ponatinib or DMSO after 24, 48, and 72 h. (**B**) Quantification of cell cycle distributions in meningioma cell lines. Each experiment was performed in triplicate and repeated three times. Results are expressed as mean ± SEM.

Next, we were interested in the effects of ponatinib on the cell cycle in meningioma cells. Therefore, ponatinib-treated cells were analyzed by flow cytometry (Figure 2A). Ponatinib induced meningioma cell cycle arrest in G0/G1-phase and subsequently increased the number of cells in the subG1-phase. After 24 h of treatment, the percentage of cells in the G0/G1-phase increased from 57.5–65.6% to 68–81.3% in all cell lines (*p* < 0.001, Figure 2B). Simultaneously, the relative number of cells in subG1-phase increased over time. Remarkably, 75.1% of NCH93 cells were in the subG1-phase compared to 3.7% in vehicle control after 72 h treatment (*p* < 0.001, Figure 2B). Furthermore, the proportion of cells in the subG1-phase also increased significantly to 44.8% and 30.0% in IOMM-Lee and Ben-Men-1 cells, respectively (*p* < 0.001, Figure 2B). Taken together, these results suggest that ponatinib blocks G1 to S-transition and subsequently an accumulation of cells in subG1-phase.

### 3.4. Ponatinib Effectively Induced Apoptosis of Meningioma Cells

**Figure 3:**
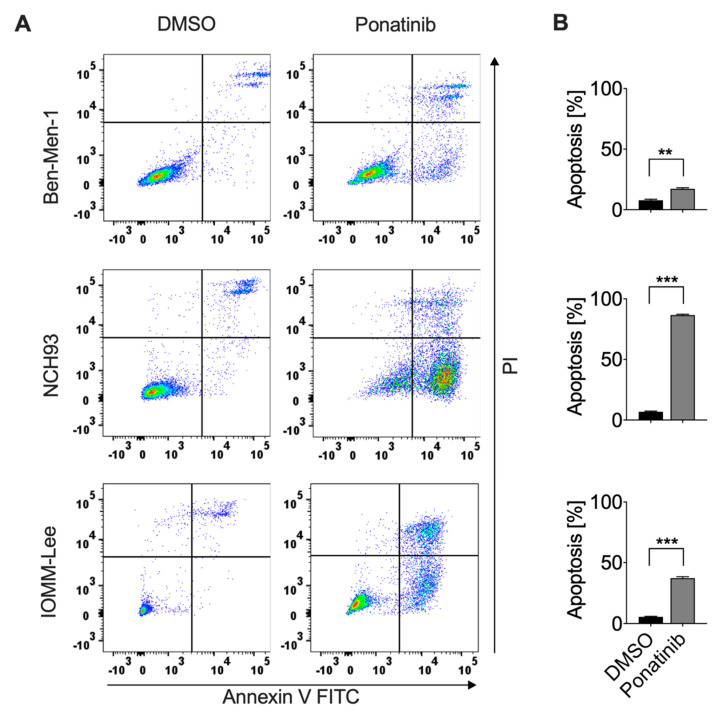
Ponatinib induced meningioma cell apoptosis: (**A**) Early- and late-stage apoptosis was induced after 72 h of treatment with 10 × IC50 of ponatinib. (**B**) Percentage of apoptotic cells in ponatinib-treated or DMSO-treated meningioma cells. The experiment was performed in triplicate and independently repeated three times. Results are expressed as mean± SEM. ** *p* < 0.01; *** *p* < 0.001.

To test whether the increased proportion in subG1-phase upon ponatinib treatment is attributed to apoptosis, ponatinib-treated meningioma cells were stained with annexin V/PI and analyzed by flow cytometry (Figure 3A). Ponatinib significantly induced apoptosis in all cell lines. In line with the previous cell cycle results, 83% of NCH93 cells underwent apoptosis after 72 h treatment with ponatinib (*p* < 0.001, Figure 3B). Furthermore, ponatinib effectively induced apoptosis up to 37.5% and 17.5% in IOMM-Lee and Ben-Men-1, respectively (*p* < 0.001; *p* = 0.0012). In summary, ponatinib-treatment leads to apoptosis in meningioma cells.

### 3.5. RTKi Ponatinib Blocks Tumor Growth In Vivo

**Figure 4:**
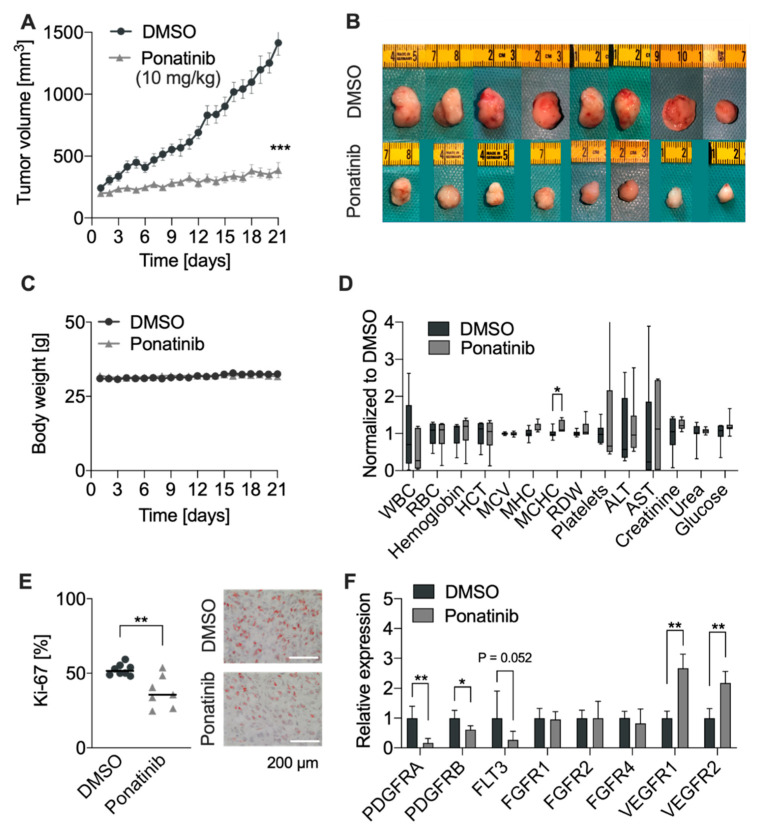
Ponatinib inhibited tumor growth in vivo: (**A**) NCH93 tumor-bearing were randomized into two groups after reaching a tumor size of 200 mm^3^. Mice were treated with ponatinib (10 mg/kg body weight, i.p. daily) for 21 days. Ponatinib significantly inhibited NCH93 tumor growth in NMRI/nu mice. (**B**) On day 21, mice were sacrificed and tumors were excised. (**C**) Mice weight was not affected by the treatment. (**D**) Blood samples from the mice were drawn immediately after killing and were then analyzed. Hematological parameters remained unchanged except for a minor change in MCHC. (**E**) Ki-67 expression indicated a significant decrease in the proliferation of ponatinib-treated tumors (left). Representative images of Ki-67 stained tumor sections (right). Bar represents 200 µm. (**F**) mRNA levels of *PDGFRA* and *PDGFRB* were reduced and mRNA levels of VEGFR1 and VEGFR2 were increased upon ponatinib treatment. * *p* < 0.05; ** *p* < 0.01; *** *p* < 0.001.

To evaluate the efficacy of ponatinib in vivo, we implanted NCH93 cells in the flank of NMRI/nu mice as described before [40,41]. After the tumor volume reached approximately 200 mm^3^, mice were randomized into ponatinib or DMSO control group. Mice were treated with 10 mg/kg bodyweight of ponatinib daily. On day 21, mice were sacrificed, weighted, drawn blood, and tumors were excised and weighted. Ponatinib potently inhibited meningioma tumor growth by 72.6% as compared to DMSO on day 21 (*p* < 0.001; Figure 4A,B). Similarly, excised tumor weights in the ponatinib-treated group were significantly lower than that in the control group (0.27 g vs. 0.94 g, *p* < 0.001, Figure S2A). The treatment had no impact on the body weight of the mice and resulted only in minor changes of blood parameters (Figure 4C,D). Only the mean corpuscular hemoglobin concentration (MCHC) was slightly affected, indicating that the applied treatment regime of ponatinib was well-tolerated in mice (*p* = 0.43, Figure 4D). Furthermore, the excised tumors were stained for the proliferation marker Ki-67 and apoptosis marker cleaved caspase-3. The immunohistochemical staining confirmed decreased levels of Ki-67 upon ponatinib treatment (ponatinib: 38%; DMSO: 52%, *p* = 0.003, Figure 4E). Interestingly, the expression of cleaved caspase-3 was less than 0.1% in both control and treatment groups, suggesting a more pronounced inhibition of proliferation rather than induction of apoptosis in vivo (Figure S2B). Moreover, we were interested whether mRNA levels were affected upon ponatinib treatment. Therefore, RNA from frozen mouse tumor samples was isolated and qRT-PCR was performed (Figure 4F). The mRNA levels of *PDGFRA* and *PDGFRB* were significantly reduced by 83.8% and 38.6% in the ponatinib-treated group (*p* = 0.0037 and *p* = 0.02, respectively). In addition, the mRNA levels of *FLT3* showed a nonsignificant reduction (*p* = 0.052). *FGFR1*, *2*, and *4* mRNA levels were not altered upon ponatinib treatment. Interestingly, the mRNA levels of *VEGFR1* and *VEGFR2* were increased significantly upon ponatinib treatment, which might be a compensatory effect. Lastly, the expressions of *FGFR3* mRNA were not detected in NCH93 xenografts. Taken together, ponatinib blocked meningioma tumor growth in vivo by decreased proliferation presumably through inhibition of *PDGFR* signaling pathway.

### 3.6. Ponatinib Treatment Induced Mitochondrial Dysfunction

**Figure 5:**
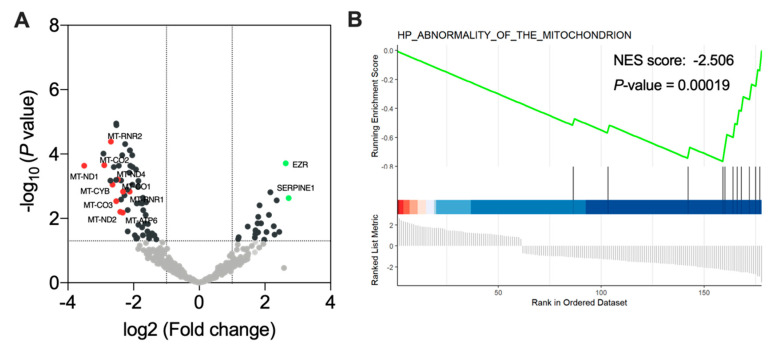
Ponatinib induced mitochondrial dysfunction: (**A**) Volcano plot of differentially expressed genes between ponatinib-treated and untreated meningiomas. Red dots: downregulated mitochondrial-related genes (log2 fold change ≤ −1; *FDR* < 0.05). Green dots: upregulated genes (log2 fold change ≥ 1; *FDR* < 0.05). (**B**) The results of gene set enrichment analysis (GESA) for GO gene sets.

To further explore the downstream effects of ponatinib treatment in meningioma, we performed full-length cDNA sequencing of ponatinib- versus DMSO-treated meningioma xenografts. By applying cutoff values of FDR < 0.05 and log2FC ≥ 1, we detected 80 differentially expressed genes, where 58 were downregulated and 22 upregulated (Table S2). Interestingly, a large proportion of the downregulated genes were mitochondrial-related, including *MT-ND1*, *MT-ND2*, *MT-ND4*, *MT-CYB*, *MT-RNR*, *MT-CO*, and *MT-ATP6* (Figure 5A). Most of these downregulated genes identified as members of the electron transport chain [42,43]. To query if mitochondria are functionally involved in the treatment response of ponatinib, we conducted gene set enrichment analysis. We discovered a significant negative enrichment of GO terms that are related to mitochondrial dysfunction (*p*_adj_ < 0.001, NES = −2.506, Figure 5B). However, when looking for changes in the receptor tyrosine kinase signaling pathway by reactome analysis, we only observed a non-significant upregulation of the signaling pathway in response to treatment (*p*_adj_ = 0.07, NES = 1.77; Figure S3). Taken together, these findings suggest that ponatinib primarily impairs the mitochondrial function, which subsequently impedes tumor growth in vivo.

## 4. Discussion

In this study, we explored the effects of the pan-TKi ponatinib on meningioma growth in vitro and in vivo. In three meningioma cell lines, proliferation was inhibited upon treatment, induced G0/G1-phase arrest and subsequently induced apoptosis. Moreover, ponatinib illustrated a significant tumor growth inhibition in vivo while being well-tolerated. Mechanistically, this was accompanied by RTK blockage of *PDGFRA*, *PDGFRB*, and *FLT3*. Furthermore, RNA-sequencing of the xenograft tumors revealed primarily deregulation of genes involved in the mitochondrial electron transport chain, suggesting mitochondrial dysfunction as an additional factor in impeding tumor growth upon ponatinib treatment.

When comparing the efficacy of ponatinib in meningioma cells to other tumor entities, its IC50 values are distributed in the lower end of the spectrum of all cancer types, ranging from 171.2 to 341.9 nM. Especially in solid tumors, reported IC50 values ranged from 20 nM up to 6.1 µM [30,31,35,36,37,44]. Only in hematological malignancies did ponatinib exert a lower effective concentration in the range of 0.3 and 12.5 nM [45]. Interestingly, when comparing IC50 values of different TKi in the same meningioma cell line, ponatinib’s IC50 value is almost 10 and 50 times lower than the corresponding IC50s of TKis regorafenib and sorafenib (0.17 μM vs. 1.5 and 7.5 μM, respectively) [19]. Similarly, the IC50 values of sunitinib were an order of magnitude higher in Ben-Men-1 and IOMM-Lee compared to ponatinib in the respective cell lines [22]. These findings indicate that ponatinib might be particularly promising for the treatment of meningioma.

To evaluate the impact of ponatinib on the cell cycle, we performed flow cytometry and annexin V staining. We found that ponatinib induced a robust G0/G1-cell cycle arrest in meningioma cells. Similar effects were reported in schwann cells and liver cancer cells [46,47]. While short-term treatment with ponatinib led to cytostasis, prolonged treatment resulted in cell death. We also observed ponatinib-induced apoptosis by accumulation of cells in subG1, which was confirmed by annexin V/PI staining. The rate of apoptosis after 72 h ponatinib treatment ranged from 17.5% to 83.0%. Similar results were also observed in rhabdomyosarcoma cells when measuring the caspase 3/7 activity [34]. Furthermore, ponatinib-treated glioblastoma U87MG cells display over 80% of condensed or fragmented nuclei upon ponatinib treatment, indicating apoptosis [36]. Collectively, these data demonstrate that ponatinib disrupts the cell cycle of meningioma cells and subsequently induces apoptosis.

To test the efficacy of ponatinib in vivo, we treated NCH93-bearing mice with a ponatinib dose of 10 mg/kg bodyweight daily. For the treatment of CML, the recommended starting dose of ponatinib is 45 mg daily [28]. Dose conversion from human to animal results in dosages of approximately 9 mg/kg body weight in mice [48]. A similar treatment regime of 10 mg/kg body weight was used by other groups [34,44,49]. The treatment resulted in significantly reduced tumor growth by 72.6% in tumor volume and 71.3% in tumor weight after 21 days. These anti-tumor effects were comparable to that of ponatinib-treated endometrial cancer xenografts, reporting a growth inhibition of 49% in mice treated with the same dose of 10 mg/kg [49]. In addition, Whittle et al. and Li et al. reported a significant reduction of tumor growth in neuroblastoma and rhabdomyosarcoma, respectively [34,44]. In addition, the proliferation rate of tumors was measured by Ki-67 staining, which showed a reduction by 14% after ponatinib treatment. Interestingly, cleaved caspase-3 staining showed less than 0.1% of apoptosis in both ponatinib-treated and control tumors, indicating that reduction in xenograft tumor growth was primarily based on a decreased proliferation. Although the heterotopic meningioma xenograft model is a well-established system to test the efficacy of drugs in mice [50], it may not entirely mirror the exact physiological tumor microenvironment and thus may induce unwanted changes in terms of proliferation, drug resistance, migration, and tumor evolution [51].

When examining the molecular targets of ponatinib in the xenograft tumors, we observed a significant reduction in mRNA levels of *PDGFRA/B* and *FLT3* upon treatment. As described from several studies, *PDGF* and its receptor expressed abundantly on meningioma cells [15,52]. Specifically, the positive rate and the immunostaining intensity of *PDGFB* and *PDGFB* receptor were higher in atypical meningiomas than in benign ones [52]. Importantly, *PDGF* has been associated with activation of two main anti-apoptotic cell signaling pathways (Ras/mitogen-activated protein kinase [MAPK] and PI3K-Akt) and one secondary pathway (phospholipase C-gamma1–protein kinase C [PLC-γ1–PKC]) simulating meningioma proliferation via autocrine and paracrine mechanisms [13,53]. Interestingly, PDGFRA expression on meningioma cells is rare and might not play an important role in meningioma pathology [52]. Collectively, these findings suggest that ponatinib exerts its effects through inhibition of PDGFRB rather than PDGFRA.

The expression of FLT3 in meningioma has been reported, however, the clinical significance in meningioma remains unclear [22]. Moreover, the mRNA levels of *VEGFR1* and *VEGFR2* were upregulated upon the treatment in vivo. It is known that the *VEGFR1* and *VEGFR2* are the targets of ponatinib, and therefore the inhibition of VEGFR by ponatinib may induce a compensatory increased transcription of the corresponding genes, which subsequently led to an upregulated mRNA level of *VEGFR1* and *VEGFR2* [28,49].

Interestingly, analysis of differentially expressed genes of sequenced xenograft tumors revealed a downregulation of mitochondrial genes and subsequent gene set enrichment analysis indicated mitochondrial dysfunction in ponatinib-treated meningiomas. These downregulated genes are vital for the assembling of mitochondrial complex I, III, IV, and V, which are the key components for the respiratory electron transport chain (ETC) [24]. The dysfunction of ETC may generate reactive oxygen species (ROS), which may subsequently induce apoptosis [54]. Several TKis such as ponatinib, sorafenib, and regorafenib have been reported to uncouple components of ETC, induce mitochondrial dysfunction, and thus promote ROS generation [25,55]. In line with our findings, ponatinib has been shown to impair the activity of mitochondrial complex I, III, and V in human hepatic HepG2 cells, and eventually impeded the function of the ETC [25]. Furthermore, in chronic eosinophilic leukemia cells, Jin et al. found that ponatinib can induce a release of apoptosis-inducing factor and cytochrome C from the mitochondria into the cytosol, which in turn triggers the mitochondrial apoptosis pathway [55]. In conclusion, ponatinib seems to negatively affect mitochondria, thus triggering cellular apoptosis.

In humans, the treatment with ponatinib is associated with some adverse events (AE), however their frequencies are low [56]; the most common AEs are cardiovascular adverse events, thrombocytopenia, abdominal pain, anemia, and rash, occurring in a dose-dependent fashion [56]. In our study, ponatinib-treated mice did not experience any weight loss during the three weeks of treatment. Moreover, blood parameters of ponatinib-treated mice were not altered except of MHCH, indicating that normal physiological functions are maintained by mice after ponatinib treatment.

## 5. Conclusions

In this work, we demonstrated that ponatinib is highly effective against meningioma in vitro and in vivo while being well-tolerated. Mechanistically, this was achieved presumably through inhibition of the PDGFRA/B-axis and mitochondrial dysfunction. Collectively, this data proposes ponatinib as a promising candidate for targeted therapy for aggressive meningioma.

## Data Availability

The data presented in this study are available in this article.

## Supplementary Materials

The following are available online at https://www.mdpi.com/article/10.3390/cancers13235898/s1, Figure S1, S2, S3. Figure S1: Ponatinib significantly inhibited the proliferation of meningioma cells, Figure S2: Ponatinib effectively inhibited the tumor growth but did not induce apoptosis in vivo, Figure S3: Gene set enrichment analysis of the receptor tyrosine kinase signaling pathway, Table S1: List of primers, Table S2: List of differentially expressed genes.
