# Supplementary material for: Receptor-Tyrosine Kinase Inhibitor Ponatinib Inhibits Meningioma Growth In Vitro and In Vivo
Source: Cancers (Basel). 2021 Nov 24;13(23):5898. doi: 10.3390/cancers13235898 (PMC8657092; doi:10.3390/cancers13235898)
Supplement: Supplementary file 1 [file cancers-13-05898-s001.zip › Supplementary Figures S1-S3.pdf]

SUPPLEMENTARY FIGURES

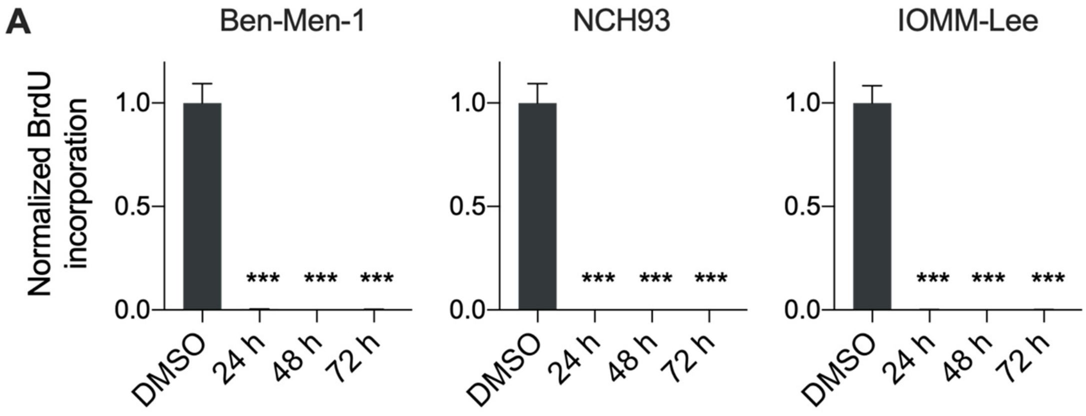

Supplementary Figure S1: Ponatinib significantly inhibited the proliferation of meningioma cells

(A) BrdU incorporation assay was performed to substantiate the anti-proliferative effects of ponatinib in meningioma cells. Cells were treated with ponatinib at 5 x IC50 concentration.

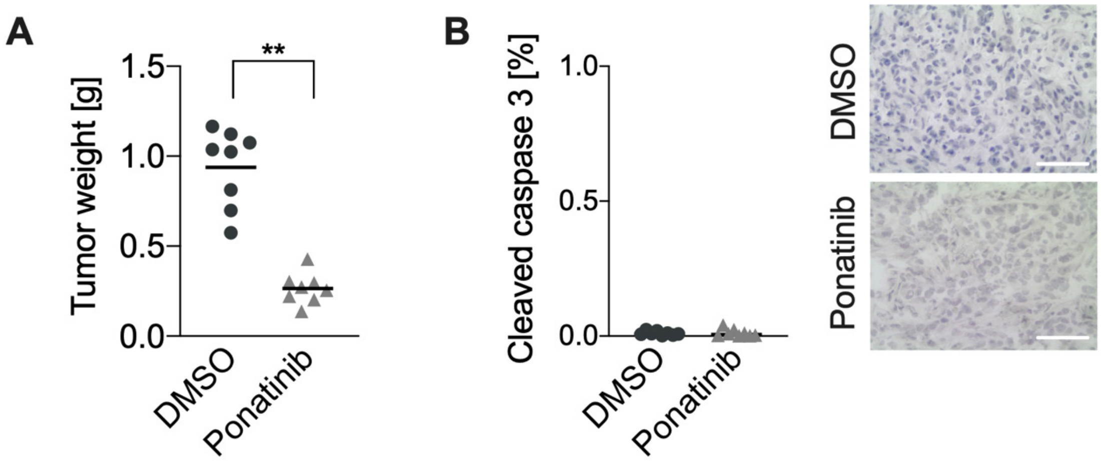

Supplementary Figure S2: Ponatinib effectively inhibited the tumor growth but did not induce apoptosis in vivo

(A) Tumor weight significantly decreased upon ponatinib treatment. **(B)** Staining of cleaved caspase-3 indicated no difference between DMSO and ponatinib-treated tumors in terms of apoptosis (left). Representative images of cleaved caspase-3 stained tumor sections (right). Bar represents 200 µm.

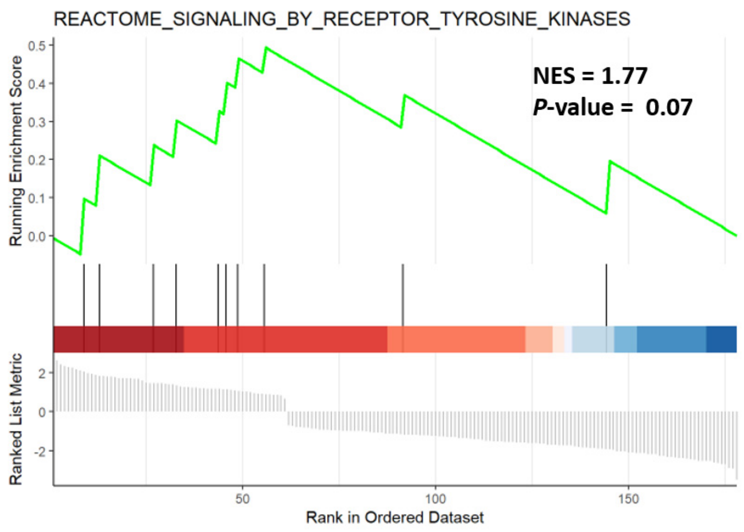

Supplementary Figure S3: Gene set enrichment analysis of the receptor tyrosine kinase signaling pathway

Reactome pathway analysis demonstrated non-significant upregulation of the RTK signaling pathway (NES = 1.77, p = 0.07).
