# Supplementary material for: Receptor-Tyrosine Kinase Inhibitor Ponatinib Inhibits Meningioma Growth In Vitro and In Vivo
Source: Cancers (Basel). 2021 Nov 24;13(23):5898. doi: 10.3390/cancers13235898 (PMC8657092; doi:10.3390/cancers13235898)
Supplement: Supplementary file 1 [file cancers-13-05898-s001.zip › Supplementary Table S1.pdf]

SUPPLEMENTARY TABLE

Supplementary Table S1: List of primers.

**Gene**		**Sequence [5'->3']**
**FLT3**	forward	TGGAATTTCTGGAATTTAAGTCG
	reverse	TTTCCCGTGGGTGACAAG
**FGFR1**	forward	ACTCCGGCCTCTATGCTTG
	reverse	AGGAGGGGAGAGCATCTGA
**FGFR2**	forward	TTCACTCTGCATGGTTGACAG
	reverse	CCCCTATGCAGTAAATGGCTA
**FGFR3**	forward	TCCTCGGGAGATGACGAA
	reverse	CAGCAGCTTCTTGTCCATCC
**FGFR4**	forward	GCCGTCAAGATGCTCAAAG
	reverse	GATCAGCTTCATCACCTCCAT
**PDGFRA**	forward	CCACCTGAGTGAGATTGTGG
	reverse	TCTTCAGGAAGTCCAGGTGAA
**PDGFRB**	forward	CATCTGCAAAACCACCATTG
	reverse	GAGACGTTGATGGATGACACC
**VEGFR1**	forward	GGCCCGGGATATTTATAAGAAC
	reverse	CCATCCATTTTAGGGGAAGTC
**VEGFR2**	forward	CCCCAAATTCCATTATGACAA
	reverse	CGGCTCTTTCGCTTACTGTT
**ACTB**	forward	CCAACCGCGAGAAGATGA
	reverse	CCAGAGGCGTACAGGGATAG
**HPRT1**	forward	TGACCTTGATTTATTTTGCATACC
	reverse	CGAGCAAGACGTTCAGTCCT
**GAPDH**	forward	AGCCACATCGCTCAGACAC
	reverse	GCCCAATACGACCAAATCC
